# Identification of Therapeutic Targets and Prognostic Biomarkers Among Chemokine (C-C Motif) Ligands in the Liver Hepatocellular Carcinoma Microenvironment

**DOI:** 10.3389/fcell.2021.748269

**Published:** 2021-12-06

**Authors:** Zhongyi Jiang, Changchang Xing, Pusen Wang, Xueni Liu, Lin Zhong

**Affiliations:** ^1^ Department of General Surgery, Shanghai General Hospital, Shanghai Jiao Tong University School of Medicine, Shanghai, China; ^2^ Department of Cardiology, Shanghai General Hospital, Shanghai Jiao Tong University School of Medicine, Shanghai, China

**Keywords:** liver hepatocellular carcinoma, biomarker, chemokine, tumor microenvironment, bioinformatics analysis

## Abstract

**Background:** Liver hepatocellular carcinoma (LIHC) is the third leading cause of cancer-related death and the sixth most common solid tumor worldwide. In the tumor microenvironment, the cross-talk between cancer cells, immune cells, and stromal cells exerts significant effects on neoplasia and tumor development and is modulated in part by chemokines. Chemokine (C-C motif) ligands (CCL) can directly target tumor cells and stromal cells, and they have been shown to regulate tumor cell proliferation, cancer stem-like cell properties, cancer invasiveness and metastasis, which directly and indirectly affect tumor immunity and influence cancer progression, therapy and patient outcomes. However, the prognostic values of chemokines CCL in LIHC have not been clarified.

**Methods:** In this study, we comprehensively analyzed the relationship between transcriptional chemokines CCL and disease progression of LIHC using the ONCOMINE dataset, GEPIA, UALCAN, STRING, WebGestalt, GeneMANIA, TRRUST, DAVID 6.8, LinkedOmics, TIMER, GSCALite, and Open Targets. We validated the protein levels of chemokines CCL through western blot and immunohistochemistry.

**Results:** The transcriptional levels of *CCL5/8/11/13/15/18/20/21/25/26/27/28* in LIHC tissues were significantly elevated while *CCL2/3/4/14/23/24* were significantly reduced. A significant correlation was found between the expression of *CCL14/25* and the pathological stage of LIHC patients. LIHC patients with low transcriptional levels of *CCL14/21* were associated with a significantly poor prognosis. The functions of differentially expressed chemokines CCL were primarily related to the chemokine signaling pathway, cytokine–cytokine receptor interactions, and TNF-α signaling pathway. Our data suggested that RELA/REL, NFKB1, STAT1/3/6, IRF3, SPI1, and JUN were key transcription factors for chemokines CCL. We found significant correlations among the expression of chemokines CCL and the infiltration of six types of immune cells (B cells, CD8^+^ T cells, CD4^+^ T cells, macrophages, neutrophils, and dendritic cells) and immune checkpoints (PD-1. PD-L1, and CTLA-4). The western blot and immunohistochemistry results showed that protein expression levels of CCL5 and CCL20 were upregulated in LIHC. CCL5 and CCL20 were significantly correlated with the clinical outcome of patients with LIHC, and could be negatively regulated by some drugs or small molecules.

**Conclusions:** Our results may provide novel insights for the potential suitable targets of immunological therapy and prognostic biomarkers for LIHC.

## Introduction

Primary liver cancer is one of the most common malignant tumors of the digestive system. According to the latest statistics from the WHO, there are 906,000 newly diagnosed cases and 830,000 deaths worldwide in 2020, making it the sixth most common tumor in the world and the third cancer-related death in humans (Sung et al., 2021). Liver hepatocellular carcinoma (LIHC) is the most important pathological type of primary liver cancer, accounting for about 75–85% (Sung et al., 2021). At present, surgical resection and liver transplantation are still the most effective strategies for the treatment of LIHC, especially for patients in the early stage of the disease with a better prognosis (Forner et al., 2018). However, due to the late clinical symptoms of LIHC and the high aggressiveness of tumors, many patients with LIHC are already at an advanced stage when they are diagnosed, and they miss the best opportunity for surgical treatment (Llovet et al., 2008). In recent years, an increasing number of studies have shown that molecular targeted therapy and immunotherapy occupy an important position in the treatment options of LIHC patients (Llovet et al., 2018; Greten et al., 2019). The liver has been recognized as an immune privilege organ (Demetris et al., 2016; Tiegs and Lohse, 2010), therefore, and identifying more therapeutic targets and prognostic biomarkers related to the tumor microenvironment is an important consideration in the current clinical management of LIHC.

The chemokine system contains genes encoding 50 chemokine ligands and 20 homologous chemokine receptors (Zlotnik and Yoshie, 2000). Chemokines and their corresponding chemokine receptors are key regulators that chemoattract immune cells and drive the inflammatory response to specific triggers (Zimmermann and Tacke, 2011). In the tumor microenvironment, the cross-talk between cancer cells, immune cells, and stromal cells exerts significant effects on neoplasia and tumor development, and chemokines can be expressed by these cells and participate in its regulation (Nagarsheth et al., 2017). A large amount of research shows that the chemokines can directly target tumor cells and stromal cells, and they have been shown to regulate tumor cell proliferation, cancer stem-like cell properties, cancer invasiveness and metastasis, which directly and indirectly affect tumor immunity and influence cancer progression, tumor therapy and patient outcomes (Atretkhany et al., 2016; Nagarsheth et al., 2017; Mollica et al., 2019). The CC (β) subfamily of chemokines, a group of chemotactic cytokines known as CCL1–28, some of which have been confirmed to be related to the development and progression of LIHC (Schneider et al., 2012; Yang et al., 2012; Zhao et al., 2021). Zhu et al. have reported that CCL14 is significantly downregulated in LIHC tumor tissues compared with peritumor tissues (Zhu et al., 2019). As a tumor suppressor, CCL14 suppressed the proliferation and promoted the apoptosis of HCC cells via inhibiting the Wnt/β-catenin-signaling pathway. Furthermore, CCL2, CCL5, and CCL7 secreted by cancer-associated fibroblasts have been shown to promote HCC metastasis by activating the hedgehog and transforming growth factor-β (TGF-β) pathway (Liu et al., 2016). Similarly, CCL15 was found to be abundantly expressed in HCC, which could recruit inflammatory monocytes in the tumor microenvironment and lead to the metastasis of HCC cells (Liu et al., 2019). Therefore, the role of chemokines CCL in the LIHC tumor microenvironment is worthy of further exploration.

Although some studies have characterized the general expression profile and functional mechanism of some chemokine CCL in LIHC, determining the appropriate chemokine CCL as a therapeutic target and prognostic biomarker of LIHC is still an enormous issue that needs urgent attention. In this study, we will use the current rapidly developing sequencing technology and various databases, combined with bioinformatics analysis, to conduct an in-depth and comprehensive discussion on the expression of chemokines CCL in LIHC, and then evaluate them as potential therapeutic targets and prognostic markers. What we have done is to provide some additional recommendations for clinically related treatment strategies of LIHC, which are conducive to the prognosis and long-term survival of patients.

## Materials and Methods

### ONCOMINE

ONCOMINE (www.oncomine.org), an online database that integrates RNA and DNA-seq from Gene Expression Omnibus (GEO), The Cancer Genome Atlas (TCGA), and published literature sources, was used to evaluate the expression of chemokines CCL in LIHC (Rhodes et al., 2004). A *p 0.05*, a fold change of 2, and a gene rank in the top 10% were set as the significance thresholds, respectively. If the data conformed to the normal distribution, the Student’s *t* test or Welch’s *t* test was used to analyze the differences in the expression of chemokines CCL in HCC, otherwise the Mann-Whitney test was used.

### GEPIA

GEPIA (Gene Expression Profiling Interactive Analysis, http://gepia.cancer-pku.cn/index.html) is currently a valuable and highly cited resource for gene expression analysis based on tumor and normal samples from TCGA and Genotype-Tissue Expression (GTEx) databases (Tang et al., 2017). In this study, based on the “LIHC” dataset, we used the single gene analysis and the multiple gene analysis module to analyze the mRNA expression levels of chemokines CCL and the prognostic correlation. The *p* value cutoff was set as 0.05. Log-rank test, a.k.a the Mantel-Cox test, and Kaplan–Meier curve were used for hypothesis test.

### UALCAN

UALCAN (http://ualcan.path.uab.edu/analysis.html) is a comprehensive, user-friendly, and interactive web resource for analyzing cancer OMICS data. It is built on PERL-CGI with high quality graphics using javascript and CSS (Chandrashekar et al., 2017). We analyzed the mRNA expression levels of chemokines CCL using the “LIHC” dataset. The student’s *t* test was used in the database analysis and the *p* value cutoff was set as 0.05.

### STRING

STRING (https://string-db.org/) is an online database for searching known protein interaction relationships, which aims to collect, score, and integrate all publicly available sources of protein–protein interaction (PPI) data, and to complement these with computational predictions of potential functions (Szklarczyk et al., 2019). We conducted a PPI network analysis of differentially expressed chemokines CCL to explore the interactions among them with STRING.

### WebGestalt

WebGestalt (WEB-based Gene SeT AnaLysis Toolkit, http://www.webgestalt.org/) is a functional enrichment analysis web tool, which has on average 26,000 unique users from 144 countries and territories per year according to Google Analytics (Liao et al., 2019). In our study, the “PPI BIOGRID” of “Network Topology-based Analysis (NTA)” method was used to analyze the subnetwork and interactions among chemokines CCL.

### GeneMANIA

GeneMANIA (http://www.genemania.org) is a flexible, user-friendly web interface for generating hypotheses about gene function, analyzing gene lists and prioritizing genes for functional assays (Warde-Farley et al., 2010).

### David 6.8

DAVID 6.8 (https://david.ncifcrf.gov/home.jsp) is a comprehensive set of functional annotation tools for investigators to understand biological meaning behind large list of genes (Huang et al., 2009). In the study, the Gene Ontology (GO) enrichment analysis and Kyoto Encyclopedia of Genes and Genomes (KEGG) pathway enrichment analysis of differentially expressed chemokines CCL and their neighboring genes were performed using DAVID 6.8. Then we visualized the results through the “ggplot2,” “dplyr,” and “stringi” packages of R project and a *p* < 0.05. Biological processes (BP), cellular components (CC), and molecular function (MF) were included in the GO enrichment analysis.

### TRRUST

TRRUST (https://www.grnpedia.org/trrust/) a manually curated database of human and mouse transcriptional regulatory networks. The current version of TRRUST contains 8,444 transcription factor (TF)-target regulatory relationships of 800 human TFs. The TRRUST database can provide information of mode of regulation (Han et al., 2018). The Fisher’s exact test and the Benjamini–Hochberg procedure were utilized in the database analysis.

### TIMER

TIMER (https://cistrome.shinyapps.io/timer/) is a comprehensive resource for systematical analysis of immune infiltrates across diverse cancer types (Li et al., 2017). In this study, “Gene module,” “Survival module,” and “Correlation module” were used to evaluate the correlation among clinical outcome and the infiltration of immune cells and aberrant expressions of chemokines CCL in patient with LIHC. The Spearman’s rho value and estimated statistical significance were also shown in the database.

### Open Targets

Open Targets provide a target-centric workflow to aid in identifying diseases potentially related to specific targets (Koscielny et al., 2017). Here, we used Open Targets to explore diseases associated with CCL5 and CCL20.

### GSCALite

GSCALite (http://bioinfo.life.hust.edu.cn/web/GSCALite/) is a web-based analysis platform for gene set cancer analysis. Genomic aberrations influence clinical responses to treatment and are potential biomarkers for drug screening (Liu et al., 2018). We integrated the drug sensitivity and gene expression profile data of cancer cell lines in Genomics of Drug Sensitivity in Cancer (GDSC) and Therapeutics Response Portal (CTRP) for research. The expression of each gene in the genome and the small molecule/drug sensitivity (IC50) were analyzed by Spearman correlation. *p* values less than 0.05 were considered statistically significant.

### Cell Culture

L02 cells, Huh7 cells, HCC-LM3 cells, HepG2 cells, and Hep3B cells were cultured using DMEM (Gibco, Thermo Fisher Scientific, Waltham, MA, USA) supplemented with 10% FBS (Gibco) and 1% penicillin-streptomycin, in 5% CO_2_ at 37°C.

### Western Blot

LIHC tissues and adjacent normal tissues were obtained at Shanghai General Hospital from 2016 to 2018 and used in our study. Written informed consent was obtained from each patient. This study was approved by ethics committee of Shanghai General Hospital. Protein extracted from tissues or cell cultures were using RIPA buffer (Thermo Fisher Scientific) mixed with PMSF (Beyotime, Shanghai, China) for 30 min on ice, and then centrifuged at 12,000 rpm for 10 min at 4°C. Protein lysates were separated using SDS-PAGE and transferred to PVDF membranes. After incubating with 5% Bovine serum albumin (BSA) for 1 h at room temperature (RT), the membranes were incubated with primary antibodies (CCL5, 1:100, AF5151; CCL20, 1: 100, DF2238) (Affinity, Changzhou, China) overnight at 4°C, washed with Tris Buffered Saline with Tween^®^20 (TBST) for 3 times, and further incubated with secondary antibodies (Sangon Biotech, Shanghai, China) for 1 h at RT, and developed using ECL solutions (Beyotime).

### Immunohistochemistry

All formalin-fixed paraffin-embedded tissues were prepared for use. After oven-drying, dewaxing, antigen retrieval, incubating with endogenous peroxidase, and blocking treatments, the slides were incubated with primary antibodies overnight at 4°C, followed by incubating with HRP-conjugated secondary antibodies at RT for 1 h. The stain was then visualized by incubation in DAB and counterstained with hematoxylin. Finally, the slides were covered with resinene and then the staining intensity was observed under the microscope (tumor tissue, *n* = 5; adjacent tissue, *n* = 5).

## Results

### Differential Expression of Chemokines CCL in Patients With LIHC

**FIGURE 1:**
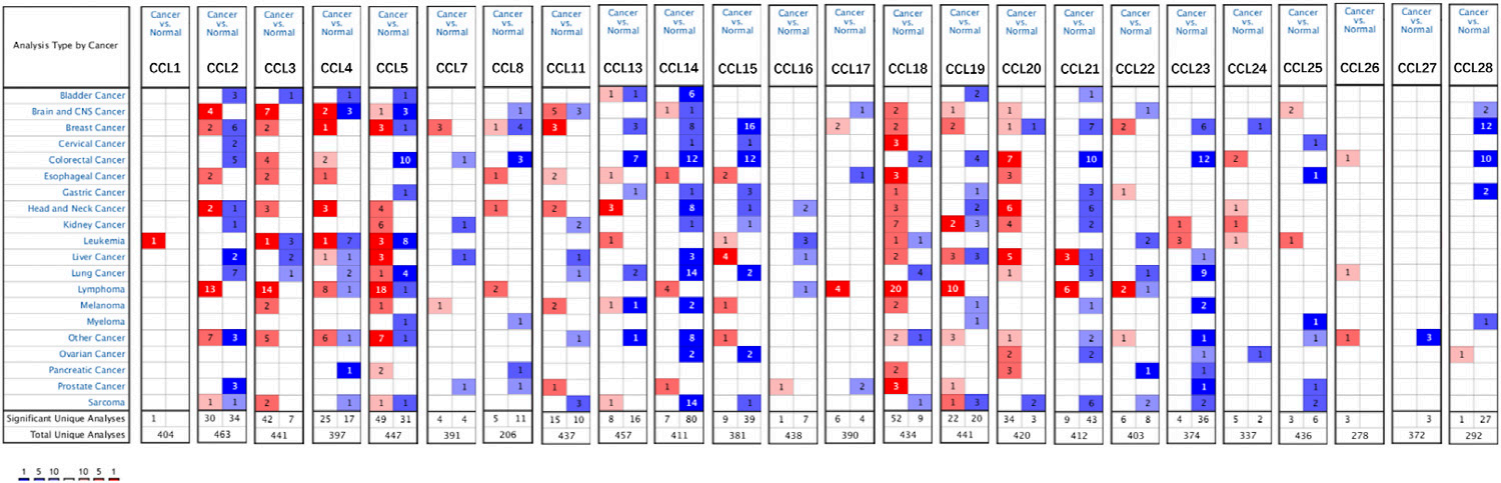
mRNA levels of chemokines CCL in LIHC (ONCOMINE). The figure shows the numbers of datasets with statistically significant mRNA upregulated (red) or downregulated expression (blue) of chemokines CCL.

Twenty-four chemokines CCL were determined using the ONCOMINE database, excluding CCL6, CCL9, and CCL10. We then explored the transcriptional levels of chemokines CCL in LIHC and compared to normal liver tissues with ONCOMINE. As shown in Figure 1 and Table 1, the transcriptional levels of *CCL5, CCL15, CCL18, CCL20,* and *CCL21* in LIHC tissues were significantly upregulated while the transcriptional levels of *CCL2, CCL3, CCL14,* and *CCL23* were significantly downregulated in LIHC compared to normal liver tissue. These results are consistent with Chen et al. who found a significant downregulation of CCL2 and CCL3 in LIHC(X. Chen et al., 2002). Wurmbach et al. also reported that the level of CCL2(*p* = 0.001) and CCL3(*p* = 0.0007) in LIHC were reduced with fold changes of −3.398 and −3.255, respectively (Wurmbach et al., 2007). Similarly, Wurmbach et al. revealed that the expression of CCL14 and CCL23 were decreased in LIHC (Wurmbach et al., 2007), which was consistent with the downregulation of CCL4 expression in the Roessler’s dataset (Roessler et al., 2010). In contrast, Mas et al. found that the transcription levels of *CCL5* and *CCL21* were significantly upregulated in LIHC (Mas et al., 2009). The fold change of CCL15 expression in LIHC was 2.168 (*p* = 0.0012) and 2.395 (*p<0.0001*) in the datasets of Wurmbach (Wurmbach et al., 2007) and Mas (Mas et al., 2009), respectively. Moreover, Wurmbach et al. suggested that CCL18 was significantly elevated in LIHC (Wurmbach et al., 2007). And The results of Wurmbach (Wurmbach et al., 2007) and Roessler (Roessler et al., 2010) all suggested that CCL20 was remarkably higher in LIHC tumors than in normal samples.

**TABLE 1:** The mRNA levels of aberrantly expressed chemokines CCL in LIHC tissues and normal liver tissues at transcriptome level (ONCOMINE).

Chemokine	Type	Fold change	*p* value	References
CCL2	LIHC	−4.256	<0.0001	Chen et al. (2002)
	LIHC	−3.398	0.001	Wurmbach et al. (2007)
CCL3	LIHC	−2.84	<0.0001	Chen et al. (2002)
	LIHC	−3.255	0.0007	Wurmbach et al. (2007)
CCL5	LIHC	3.596	<0.0001	Mas et al. (2009)
CCL14	LIHC	−5.613	<0.0001	Wurmbach et al. (2007)
	LIHC	−3.643	<0.0001	Roessler et al. (2010)
CCL15	LIHC	2.168	0.0012	Wurmbach et al. (2007)
	LIHC	2.395	<0.0001	Mas et al. (2009)
CCL18	LIHC	2.504	<0.0001	Wurmbach et al. (2007)
CCL20	LIHC	6.933	0.002	Wurmbach et al. (2007)
	LIHC	9.778	<0.0001	Roessler et al. (2010)
CCL21	LIHC	3.093	<0.0001	Mas et al. (2009)
CCL23	LIHC	−2.148	<0.0001	Wurmbach et al. (2007)

**FIGURE 2:**
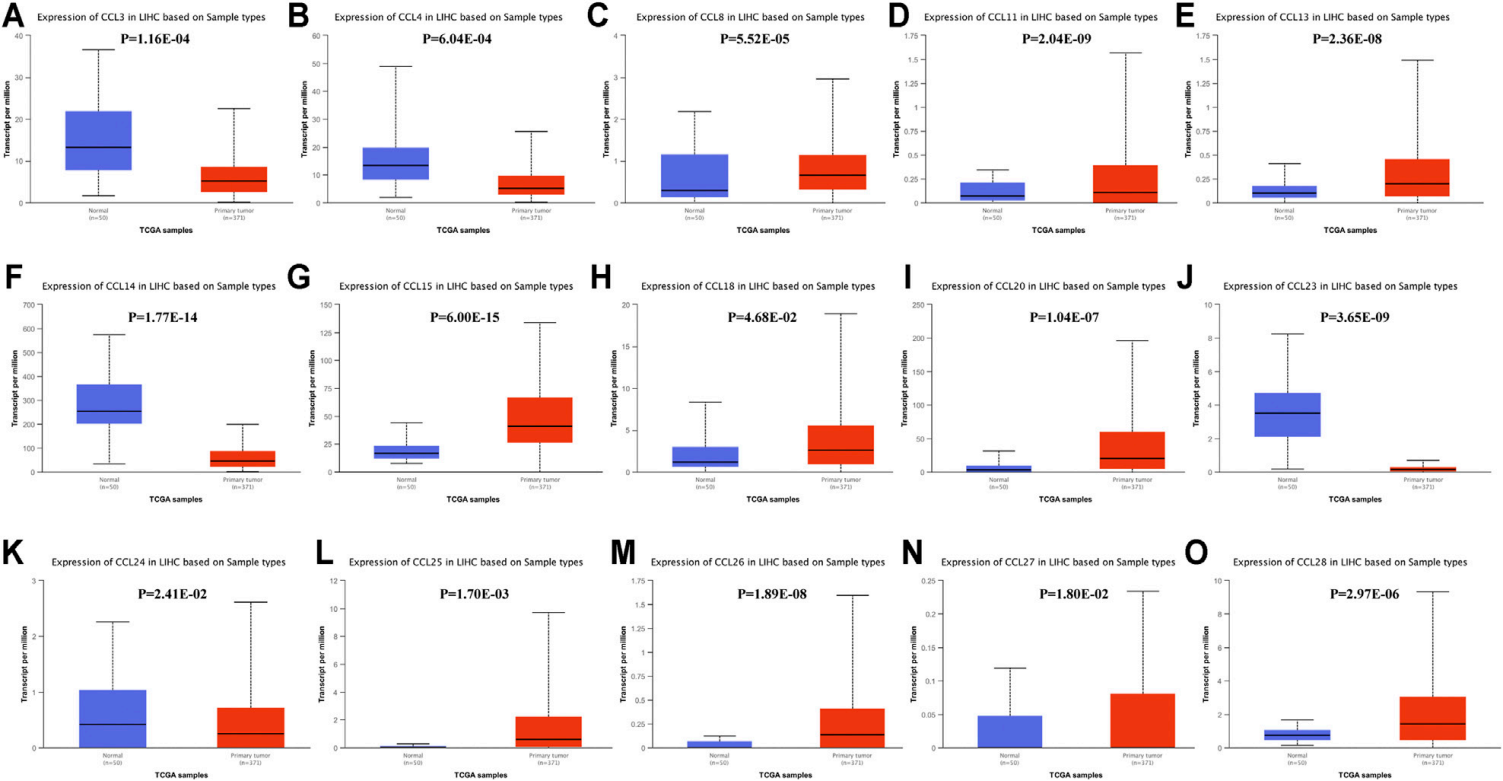
The transcription levels of chemokines CCL in LIHC (UALCAN). The transcriptional levels of **(C)** CCL8, **(D)** CCL11, **(E)** CCL13, **(G)** CCL15, **(H)** CCL18, **(I)** CCL20, **(L)** CXCL25, **(M)** CCL26, **(N)** CCL27, and **(O)** CCL28 in RCC tissues were significantly elevated while the transcriptional levels of **(A)** CCL3, **(B)** CCL4, **(F)** CCL14, **(J)** CCL23, and **(K)** CCL24 were remarkably reduced. *p* < 0.05 was considered statistically significant.

We also used the UALCAN database to evaluate the expression levels of chemokines CCL in LIHC tissues and normal liver tissues. Consistent with the ONCOMINE data, the transcriptional levels of *CCL3*(*p* = 1.16E-04), *CCL14*(*p* = 1.77E-14), and *CCL23*(*p* = 3.65E-09) were significantly downregulated in LIHC compared to normal liver tissue, while the transcriptional levels of *CCL15*(*p* = 6.00E-15), *CCL18*(*p* = 4.68E-02), and *CCL20*(*p* = 1.04E-07) were significantly upregulated (Figure 2). In addition, the results of the UALCAN database showed that CCL4(*p* = 6.04E-04) and CCL24(*p* = 2.41E-02) were reduced remarkably and CCL8(*p* = 5.52E-05), CCL11(*p* = 2.04E-09), CCL13(*p* = 2.36E-08), CCL25(*p* = 1.70E-03), CCL26(*p* = 1.89E-08), CCL27(*p* = 1.80E-02), and CCL28(*p* = 2.97E-06) were elevated significantly in LIHC tumors compared with normal liver samples. Combining the results of the two databases, we have excluded those chemokines CCL whose expressions were not significantly different, including CCL1, CCL7, CCL12, CCL16, CCL17, CCL19, and CCL22.

**FIGURE 3:**
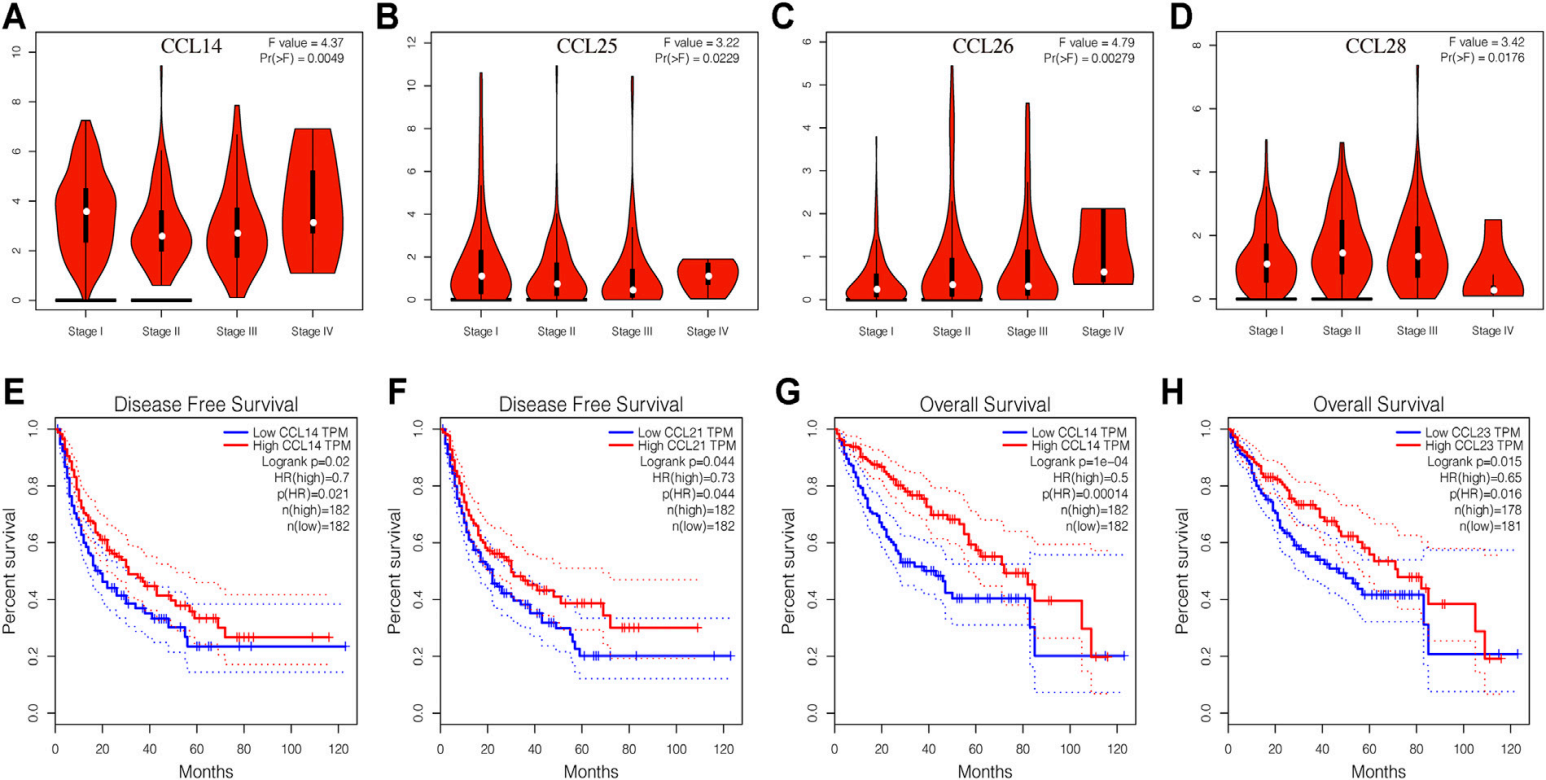
Correlation between aberrantly expressed chemokines CCL and the pathological stage, DFS, and OS of LIHC patients (GEPIA). **(A–D)** Correlation between the pathological stage and expression of CCL14, CCL25, CCL26, and CCL28, respectively. **(E,F)** The prognostic value of expression of CCL14 and CCL21 in LIHC patients in the DFS curve. **(G,H)** The prognostic value of expression of CCL14 and CCL23 in LIHC patients in the OS curve. *p* < 0.05 was considered statistically significant.

To identify chemokines CCL associated with tumorigenesis and progression in LIHC, we further explored the correlation between the expression of differentially expressed chemokines CCL and the pathological stage of LIHC patients. As expected, the expression of CCL14, CCL25, CCL26, and CCL28 were obviously related to the pathological stage (Figures 3A–D), and with the aggravation of tumor malignancy, the expression of CCL25, CCL26, and CCL28 were increased while CCL14 was decreased. As we have seen, there is a certain correlation between the expression of chemokines CCL and tumor progression of LIHC.

### The Prognostic Value of Chemokines CCL in Patients With LIHC

We then used the GEPIA database to evaluate the correlation between the expression of differentially expressed chemokines CCL and the clinical prognosis of LIHC patients. As shown in Figures 3E,F, the high transcription levels of *CCL14* (*p* = 0.021) and *CCL21* (*p* = 0.044) in LIHC patients were significantly associated with better disease-free survival (DFS). More importantly, we also proved that LIHC patients with high *CCL14* **(** Figure 3G **)** and *CCL23* **(** Figure 3H **)** transcription levels have longer overall survival (OS).

### PPI Network, and Interaction Analyses of Chemokines CCL in Patients With LIHC

**FIGURE 4:**
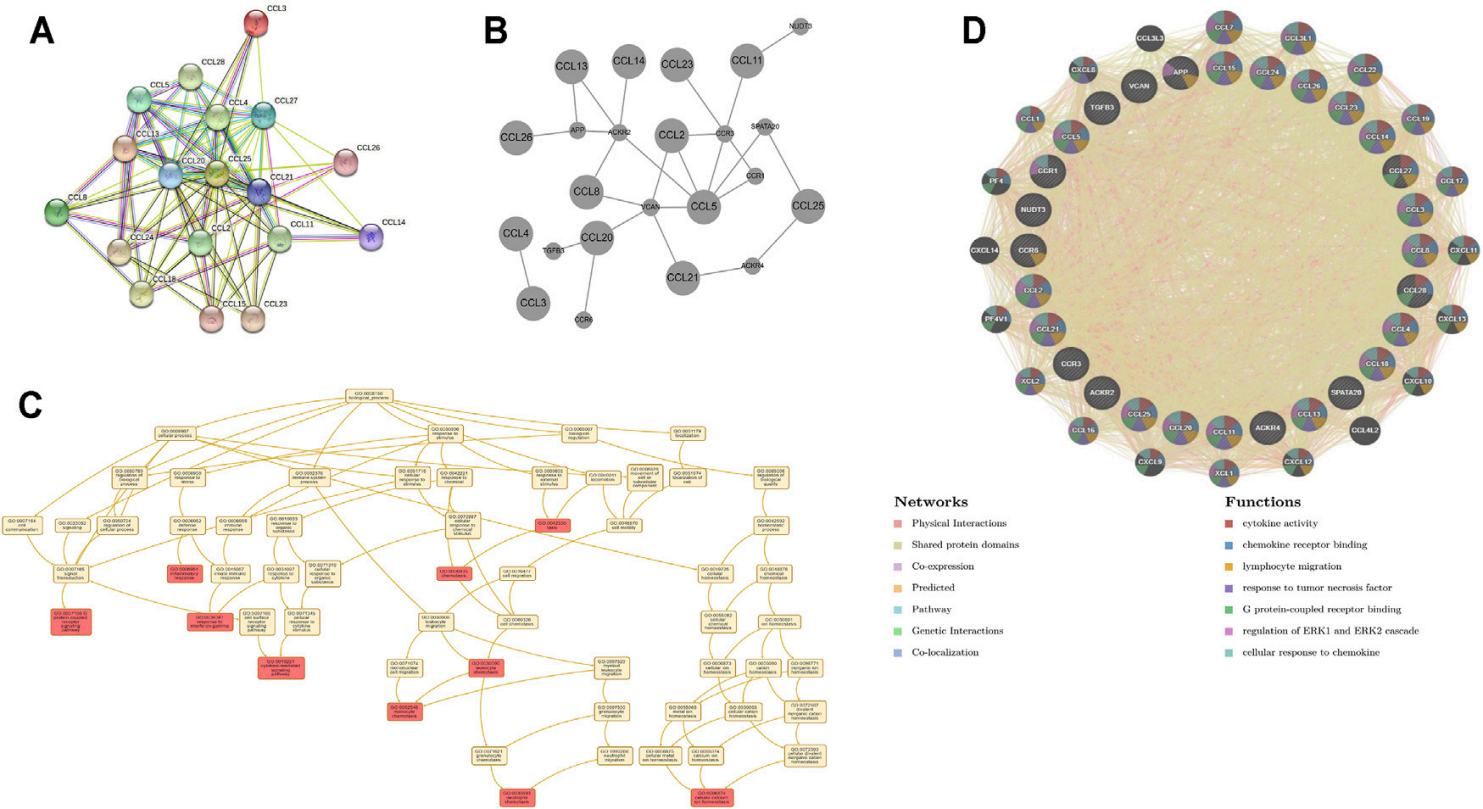
PPI network, neighboring gene network, interaction analyses and functional enrichment of aberrantly expressed chemokines CCL in LIHC patients. **(A)** PPI network of aberrantly expressed chemokines CCL. **(B)** Gene–gene interaction network of aberrantly expressed chemokines CCL and their most frequently altered neighboring genes. **(C)** GO enrichment of chemokines CCL and their neighboring genes. **(D)** PPI network and functional enrichment of chemokines CCL and their neighboring genes.

To better understand the potential interactions between differentially expressed chemokines CCL, we used the STRING database to construct a PPI network (Figure 4A). The result revealed that there are 18 nodes and 84 edges in the PPI network. The functional enrichments in the network showed that these differentially expressed chemokines CCL was associated with the chemokine signaling pathway and the NF-κB signaling pathway. In addition, we further used WebGestalt to verify the interaction relationship of differentially expressed chemokines CCL (Figure 4B). The Top Ranking Neighbors associated with differentially expressed chemokines CCL including APP, ACKR2, VCAN, ACKR4, CCR3, SPATA20, NUDT3, CCR1, TGFB3, and CCR6. Moreover, GO enrichment analysis showed that the differential expression of chemokines CCL and their neighboring genes were related to chemotaxis and inflammatory response, G protein-coupled receptor signaling pathway, and cytokine-mediated signaling pathway (Figure 4C; Table 2). Similarly, GeneMANIA analysis demonstrated that the functions of chemokines CCL and their neighboring genes were mainly associated with cytokine activity, chemokine receptor binding, lymphocyte migration, response to tumor necrosis factor, etc. (Figure 4D).

**TABLE 2:** The GO functional enrichment of chemokines CCL and their neighboring genes (WebGestalt).

GO ID	GO Name	Size	Overlap	Raw *p* value	Adjusted *p* value	Interest Gene
GO:0002548	monocyte chemotaxis	51	13	0	0	CCR1; CCL2; CCL3; CCL5; CCL8; CCL11; CCL13; CCL14; CCL20; CCL21; CCL23; CCL25; CCL26
GO:0006874	cellular calciumion homeostasis	391	14	0	0	APP; CCR1; CCR3; CCR6; ACKR2; CCL3; CCL5; CCL8; CCL11; CCL13; CCL14; CCL21; CCL23; ACKR4
GO:0006935	chemotaxis	551	18	0	0	APP; CCR1; CCR3; CCR6; ACKR2; CCL2; CCL3; CCL5; CCL8; CCL11; CCL13; CCL14; CCL20; CCL21; CCL23; CCL25; CCL26; ACKR4
GO:0006954	Inflammatory response	656	17	0	0	APP; CCR1; CCR3; CCR6; ACKR2; CCL2; CCL3; CCL5; CCL8; CCL11; CCL13; CCL14; CCL20; CCL21; CCL23; CCL25; CCL26
GO:0007186	G protein-coupled receptor signaling pathway	751	18	0	0	APP; CCR1; CCR3; CCR6; ACKR2; CCL2; CCL3; CCL5; CCL8; CCL11; CCL13; CCL14; CCL20; CCL21; CCL23; CCL25; CCL26; ACKR4
GO:0019221	cytokine-mediated signaling pathway	661	17	0	0	CCR1; CCR3; CCR6; ACKR2; CCL2; CCL3; CCL5; CCL8; CCL11; CCL13; CCL14; CCL20; CCL21; CCL23; CCL25; CCL26; ACKR4
GO:0030593	neutrophil chemotaxis	88	12	0	0	CCL2; CCL3; CCL5; CCL8; CCL11; CCL13; CCL14; CCL20; CCL21; CCL23; CCL25; CCL26
GO:0030595	leukocyte chemotaxis	188	14	0	0	CCR1; CCR6; CCL2; CCL3; CCL5; CCL8; CCL11; CCL13; CCL14; CCL20; CCL21; CCL23; CCL25; CCL26
GO:0034341	response to interferon-gamma	177	12	0	0	CCL2; CCL3; CCL5; CCL8; CCL11; CCL13; CCL14; CCL20; CCL21; CCL23; CCL25; CCL26
GO:0042330	taxis	553	18	0	0	APP; CCR1; CCR3; CCR6; ACKR2; CCL2; CCL3; CCL5; CCL8; CCL11; CCL13; CCL14; CCL20; CCL21; CCL23; CCL25; CCL26; ACKR4

### Transcription Factor Targets of Chemokines CCL in Patients With LIHC

Since the expression of chemokines CCL in LIHC tissues and normal liver tissues was significantly different, TRRUST was utilized to study the potential transcription factor targets of the differentially expressed chemokines CCL. All differentially expressed chemokines CCL were included in the TRRUST analysis, however, only 9 genes were involved, including *CCL2, CCL3, CCL4, CCL5, CCL11, CCL13, CCL20, CCL21,* and *CCL26*. This finding indicated that a total of 9 transcription factors (RELA, REL, NFKB1, STAT1/3/6, IRF3, SPI1, and JUN) were involved in the transcriptional regulation of chemokines CCL (Table 3). Among them, RELA and NFKB1 were pivotal transcription factors for *CCL2, CCL3, CCL4, CCL5, CCL11, CCL13,* and *CCL20*. Furthermore, REL, IRF3, SPI1, and JUN were pivotal transcription factors for *CCL2* and *CCL5.* STAT1, STAT 3, STAT6 were the key transcription factors for *CCL2* and *CCL3*, *CCL2* and *CCL11*, *CCL11* and *CCL26*, respectively.

**TABLE 3:** Key regulated factor of chemokines CCL in LIHC (TRRUST).

Key TF	Description	Regulated gene	*p* Value	FDR
RELA	v-rel reticuloendotheliosis viral oncogene homolog A (avian)	CCL4, CCL20, CCL2, CCL3, CCL11, CCL5, CCL13	6.69E-09	3.5E-08
NFKB1	nuclear factor of kappa light polypeptide gene enhancer in B-cells 1	CCL5, CCL3, CCL20, CCL11, CCL13, CCL4, CCL2	7.00E-09	3.5E-08
IRF3	interferon regulatory factor 3	CCL2, CCL5	8.96E-05	0.000299
REL	v-rel reticuloendotheliosis viral oncogene homolog (avian)	CCL2, CCL5	0.000196	0.000491
STAT6	signal transducer and activator of transcription 6, interleukin-4 induced	CCL11, CCL26	0.000531	0.00106
SPI1	spleen focus forming virus (SFFV) proviral integration oncogene spi1	CCL2, CCL5	0.00157	0.00262
STAT1	signal transducer and activator of transcription 1, 91 kDa	CCL2, CCL3	0.00286	0.00409
STAT3	signal transducer and activator of transcription 3 (acute-phase response factor)	CCL11, CCL2	0.00795	0.00969
JUN	jun proto-oncogene	CCL5, CCL2	0.00872	0.00969

### Immune Cell Infiltration of Chemokines CCL in Patients With LIHC

**FIGURE 5:**
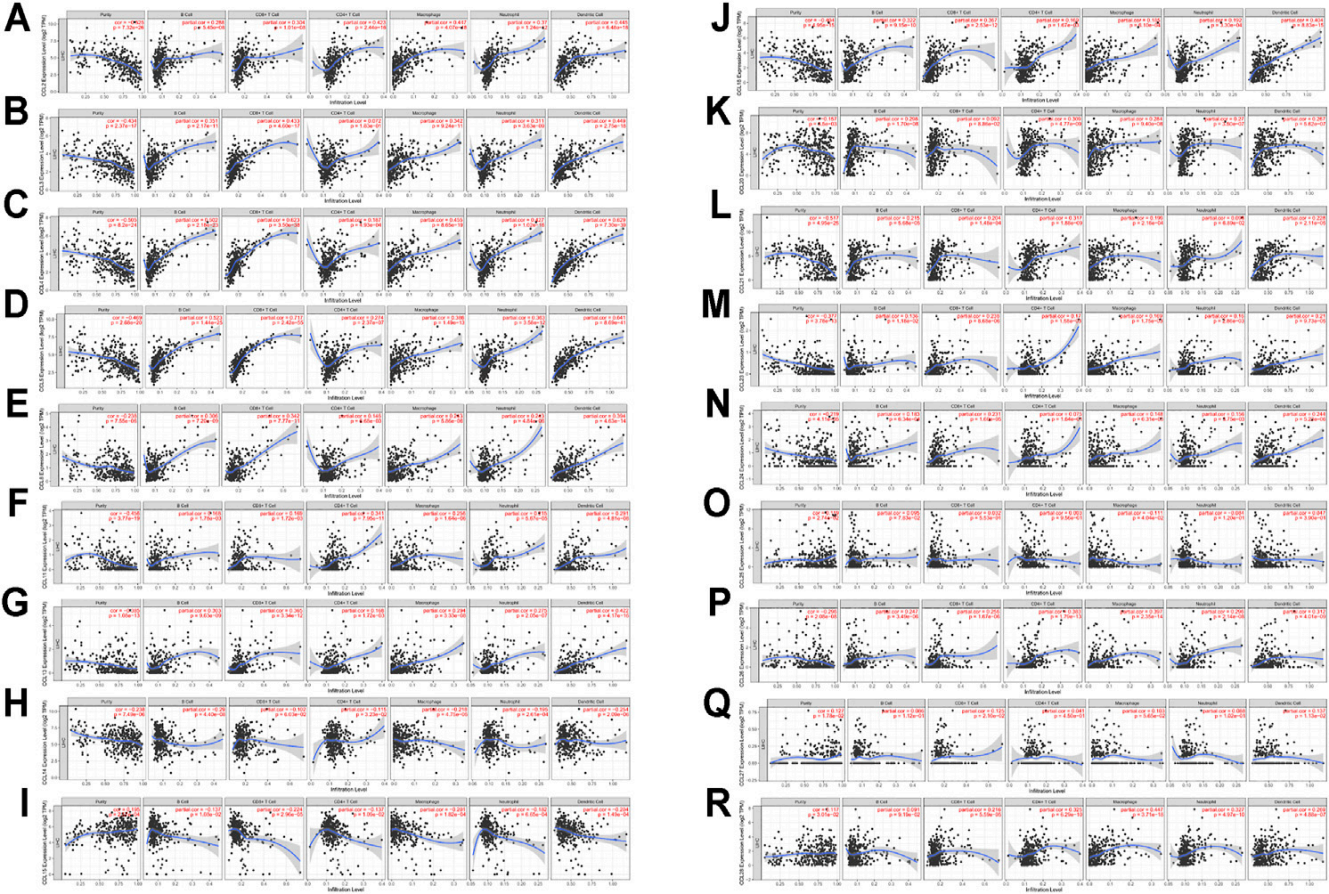
The correlation between aberrantly expressed chemokines CCL and immune cell infiltration (TIMER). The correlation between the numerous of immune cell and the expression of **(A)** CCL2, **(B)** CCL3, **(C)** CCL4, **(D)** CCL5, **(E)** CCL8, **(F)** CCL11, **(G)** CCL13, **(H)** CCL14, **(I)** CCL15, **(J)** CCL18, **(K)** CCL20, **(L)** CCL21, **(M)** CCL23, **(N)** CCL24, and **(O)** CCL25, **(P)** CCL26, **(Q)** CCL27, and **(R)** CCL28 in LIHC.

In the tumor microenvironment, chemokines CCL are related to the inflammatory response and the regulation of immune cells, which affect the prognosis of patients with LIHC. Therefore, regarding the association between differential expression of chemokines CCL and immune cell infiltration, we performed a comprehensive exploration using the TIMER database. The expression of CCL2, CCL4, CCL5, CCL8, CCL11, CCL13, CCL18, CCL23, and CCL26 were positively associated with the infiltration of B cells, CD8^+^ T cells, CD4^+^ T cells, macrophages, neutrophils and dendritic cells (Figures 5A,C–G,J,M,P), while the expression of CCL15 was negatively associated with the infiltration of B cells, CD8^+^ T cells, CD4^+^ T cells, macrophages, neutrophils and dendritic cells (Figure 5I **)**. The expression of CCL3 and CCL24 were positively associated with the infiltration of B cells, CD8^+^ T cells, macrophages, neutrophils and dendritic cells (all *p* < 0.05; Figures 5B,N **)**. CCL14 expression was negatively associated with the infiltration of B cells, CD4^+^ T cells, macrophages, neutrophils and dendritic cells (all *p* < 0.05; Figure 5H **)**. In contrast, CCL20 expression was positively associated with the infiltration of B cells, CD4^+^ T cells, macrophages, neutrophils and dendritic cells (all *p* < 0.05; Figure 5K **)**. In addition, there was a positive correlation between CCL21 expression and the infiltration of B cells, CD8^+^ T cells, CD4^+^ T cells, macrophages, and dendritic cells (all *p* < 0.05; Figure 5L **)**. CCL25 expression was negatively associated with the infiltration of macrophages (Cor = −0.111, *p* = 4.04e−2; Figure 5O **)**. CCL27 expression was positively associated with the infiltration of CD8^+^ T cells (Cor = 0.125, *p* = 2.10e−2) and dendritic cells (Cor = 0.137, *p* = 1.13e−2; Figure 5Q **)**. Except for B cells, the expression of CCL28 was positively correlated with the infiltration of CD8^+^ T cells, CD4^+^ T cells, macrophages, neutrophils and dendritic cells (all *p* < 0.05; Figure 5R **)**. We also assessed the association of differentially expressed chemokines CCL and immune cells infiltration using multivariable Cox proportional hazard model. As shown in Table 4, B cells (*p* = 0.001), macrophages (*p* = 0.005), dendritic cells (*p* = 0), CCL2 expression (*p* = 0.04), CCL5 expression (*p* = 0.01), and CCL20 expression (*p* = 0.017) were significantly correlated with the clinical outcome of patients with LIHC.

**TABLE 4:** The cox proportional hazard model of chemokines CCL and six tumor-infiltrating immune cells in LIHC (TIMER).

	Coef	HR	95%CI_l	95% CI_u	p.value	Sig
B_cell	−13.46	0	0	0.003	0.001	**
CD8_Tcell	−1.275	0.279	0.001	138.054	0.687	
CD4_Tcell	−1.064	0.345	0	380.171	0.766	
Macrophage	7.45	1719.249	9.291	318,144.1	0.005	**
Neutrophil	−2.996	0.05	0	4,447.167	0.606	
Dendritic	7.232	1,383.039	27.768	68,884.11	0	***
CCL2	−0.235	0.79	0.631	0.99	0.04	*
CCL3	0.026	1.026	0.711	1.482	0.89	
CCL4	−0.028	0.972	0.622	1.521	0.902	
CCL5	−0.362	0.697	0.53	0.916	0.01	*
CCL8	0.319	1.376	0.873	2.169	0.169	
CCL11	−0.17	0.844	0.549	1.297	0.439	
CCL13	−0.007	0.993	0.722	1.366	0.965	
CCL14	−0.117	0.89	0.737	1.074	0.224	
CCL15	0.046	1.047	0.885	1.239	0.595	
CCL18	−0.065	0.937	0.801	1.097	0.421	
CCL20	0.108	1.114	1.019	1.217	0.017	*
CCL21	0.042	1.043	0.905	1.201	0.562	
CCL23	0.023	1.023	0.541	1.933	0.945	
CCL24	0.07	1.073	0.834	1.38	0.584	
CCL25	0.009	1.009	0.923	1.103	0.845	
CCL26	0.103	1.108	0.897	1.369	0.34	
CCL27	0.391	1.479	0.347	6.301	0.597	
CCL28	−0.044	0.957	0.801	1.144	0.63	

*p < 0.05, **p < 0.01, ***p < 0.001.

### Correlation Between Immune Checkpoints and Chemokines CCL in Patients With LIHC

**FIGURE 6:**
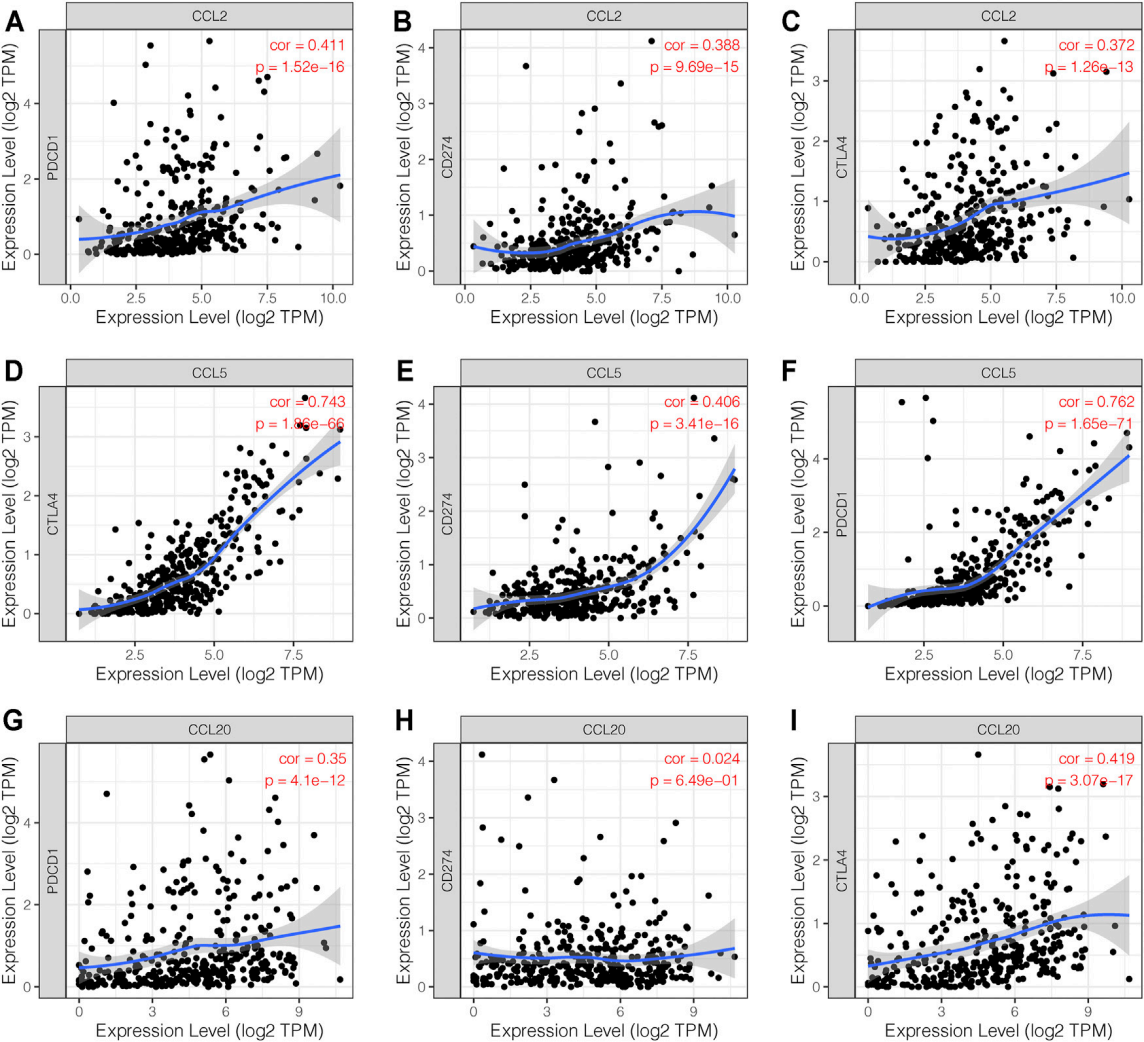
The correlation between chemokines CCL and immune checkpoints (TIMER). **(A–F)** The expression levels of CCL2 and CCL5 were positively associated with PD-1, PD-L1, and CTLA-4. **(G–I)** The expression level of CCL20 was positively associated with PD-1 and CTLA-4.

In order to explore the association between the chemokines CCL and immune checkpoints, we performed Correlation module analysis using the TIMER database. Based on the multivariable Cox proportional hazard model, we found that CCL2 and CCL5 were positively correlated with programmed cell death protein 1 (PD-1, also called PDCD1), programmed cell death-Ligand 1 (PD-L1, also called CD274), and cytotoxic T-lymphocyte-associated protein 4 (CTLA-4). In addition, CCL20 was also positively associated with PD-1 and CTLA-4 **(** Figure 6).

### Functional Enrichment Analysis of Chemokines CCL in Patients With LIHC

**FIGURE 7:**
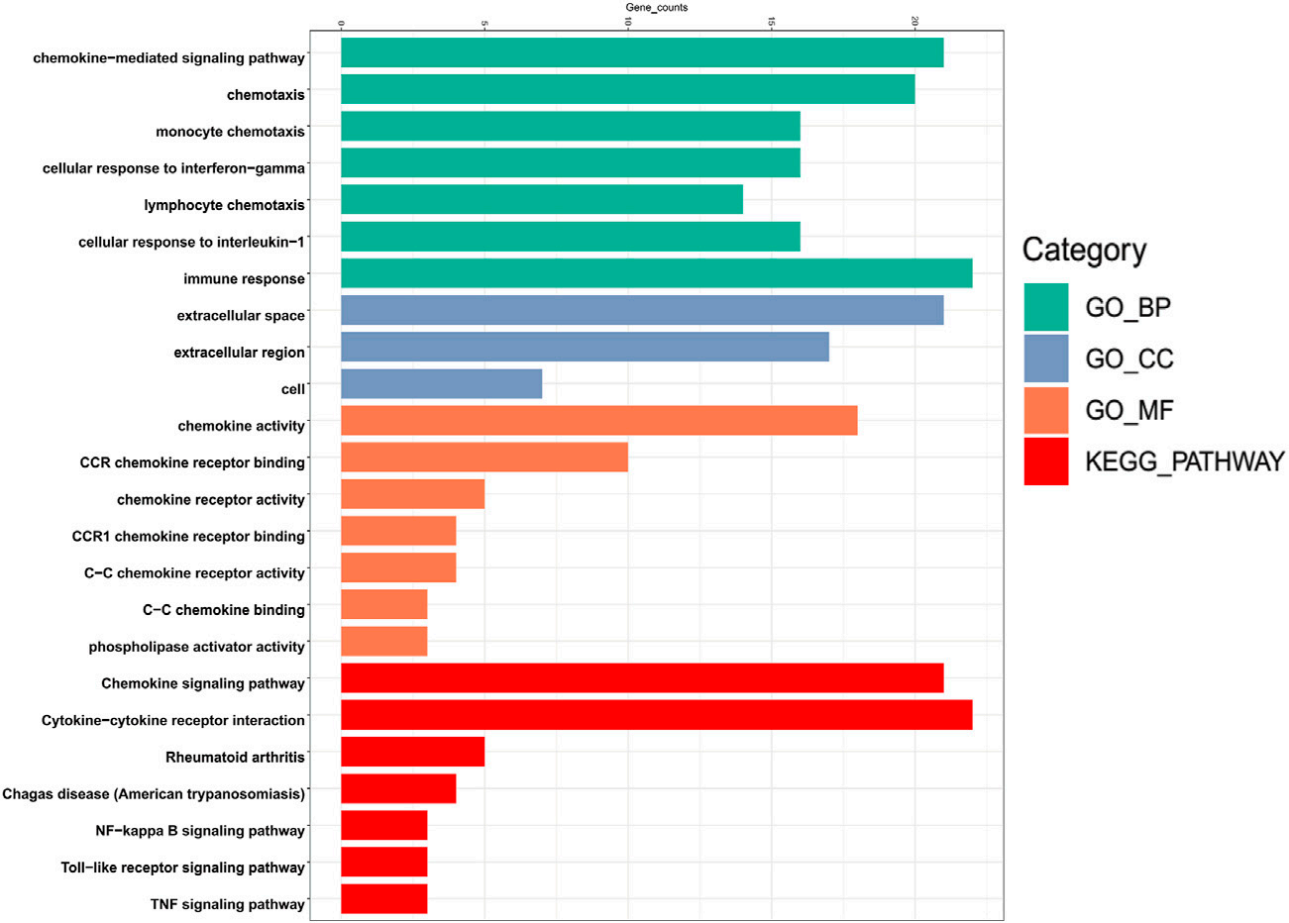
Bar plot of GO enrichment in cellular component terms, biological process terms, molecular function terms, and KEGG enriched terms of aberrantly expressed chemokines CCL and their most frequently altered neighboring genes in LIHC (David 6.8).

To explore the specific mechanism of differentially expressed chemokines CCL and their neighboring genes in LIHC, we used DAVID 6.8 for functional enrichment analysis, which makes the most meaningful enrichment based on the significance of the *p* value (Figure 7 **)**. In the results, the GO BP category indicated that the disease progression of LIHC was related to chemokine-mediated signaling pathway, chemotaxis, cellular response to interferon-gamma, cellular response to interferon-1, and immune response. The extracellular space, extracellular region, and cell were the most highly enriched items in the GO CC category. Furthermore, the chemokine activity and chemokine receptor binding were primarily enriched items in the GO MF category. In addition, in the enrichment analysis of the KEGG pathway, the differentially expressed chemokines CCL and their neighboring genes were mainly enriched in chemokine signaling pathway, cytokine-cytokine receptor interaction, nuclear factor kappa B (NF-κB) signaling pathway, toll-like receptor (TLR) signaling pathway, and tumor necrosis factor (TNF) signaling pathway. It was worth noting that CCL5, and CCL20 were key regulators of the TNF signaling pathway.

### CCL5 and CCL20 Were Upregulated in Both HCC Cell Lines and Tumor Tissues

**FIGURE 8:**
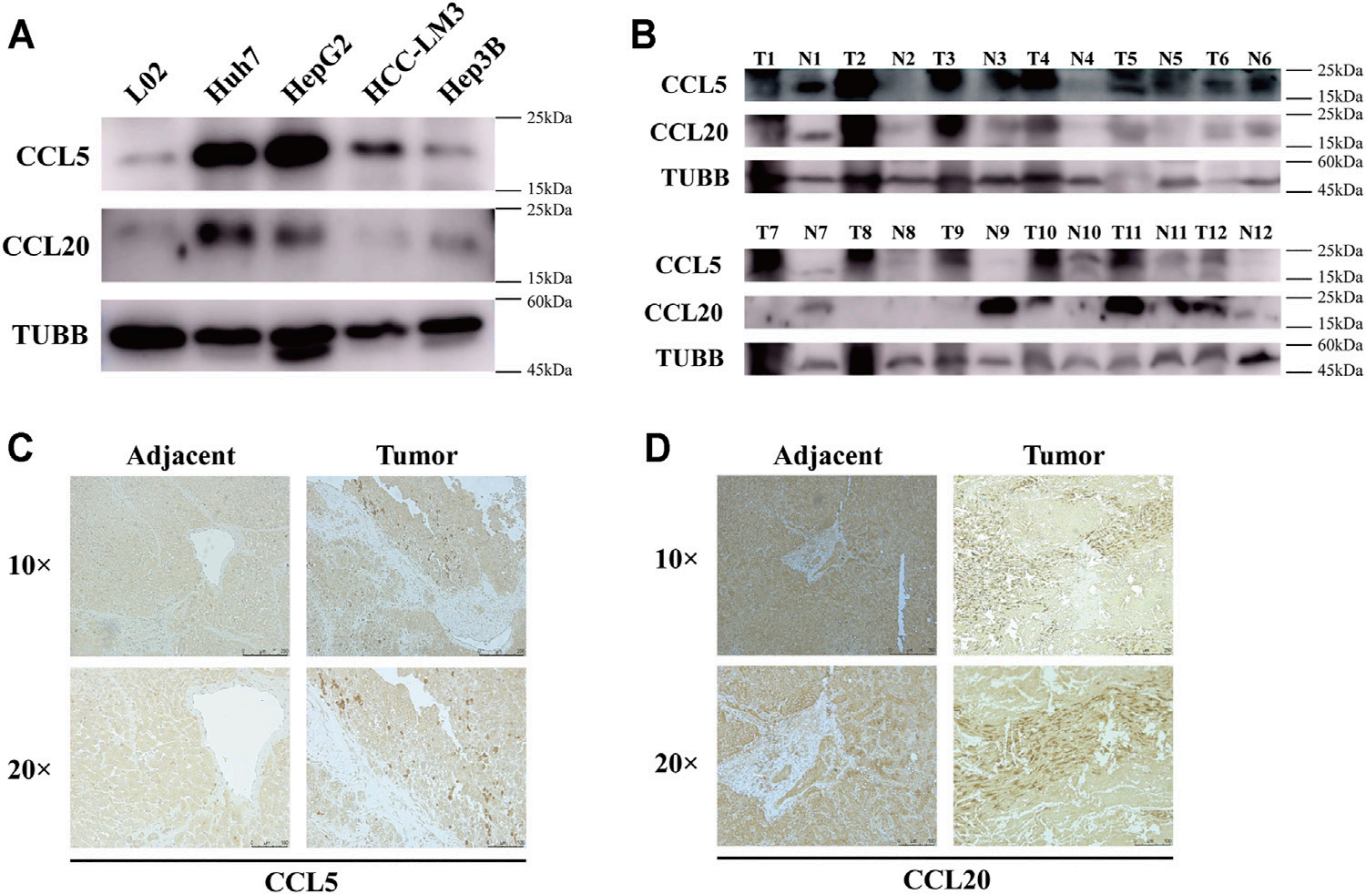
CCL5 and CCL20 expressions are increased in HCC cell lines and tumor tissues. **(A)** The protein levels of CCL5 and CCL20 in 4 HCC cell lines and human liver normal cell line determined by Western blot; TUBB was used as a control. **(B)** The protein levels of CCL5 and CCL20 in 12 HCC tissues and paired adjacent tissues determined by Western blot. **(C)** Representative images of CCL5 immunohistochemical staining in HCC tissues and paired adjacent tissues. Scale bar = 250 and 100 μm, respectively. **(D)** Representative images of CCL20 immunohistochemical staining in HCC tissues and paired adjacent tissues. Scale bar = 250 and 100 μm, respectively.

To test the protein expression levels of CCL5 and CCL20, we performed western blot and immunohistochemistry. As expected, CCL5 was dramatically upregulated in HCC cell lines, including Huh7, HepG2, HCC-LM3, and Hep3B, compared to human liver normal cell line L02 (Figure 8A **)**. Similarly, the expression level of CCL20 was also significantly increased in Huh7, HepG2, and Hep3B cells compared to L02 cells (Figure 8A **)**. Furthermore, the results of tissue samples have demonstrated that both CCL5 and CCL20 were markedly upregulated in HCC tumor tissues than in adjacent tissues (Figure 8B **)**. Coincidentally, we also showed that the expressions of CCL5 and CCL20 were appreciably increased in HCC tumor tissues compared to adjacent tissues by immunohistochemistry (Figures 8C,D **)**.

### Disease Susceptibility and Drug Sensitivity Analysis of Aberrant Expression of Chemokines CCL

**FIGURE 9:**
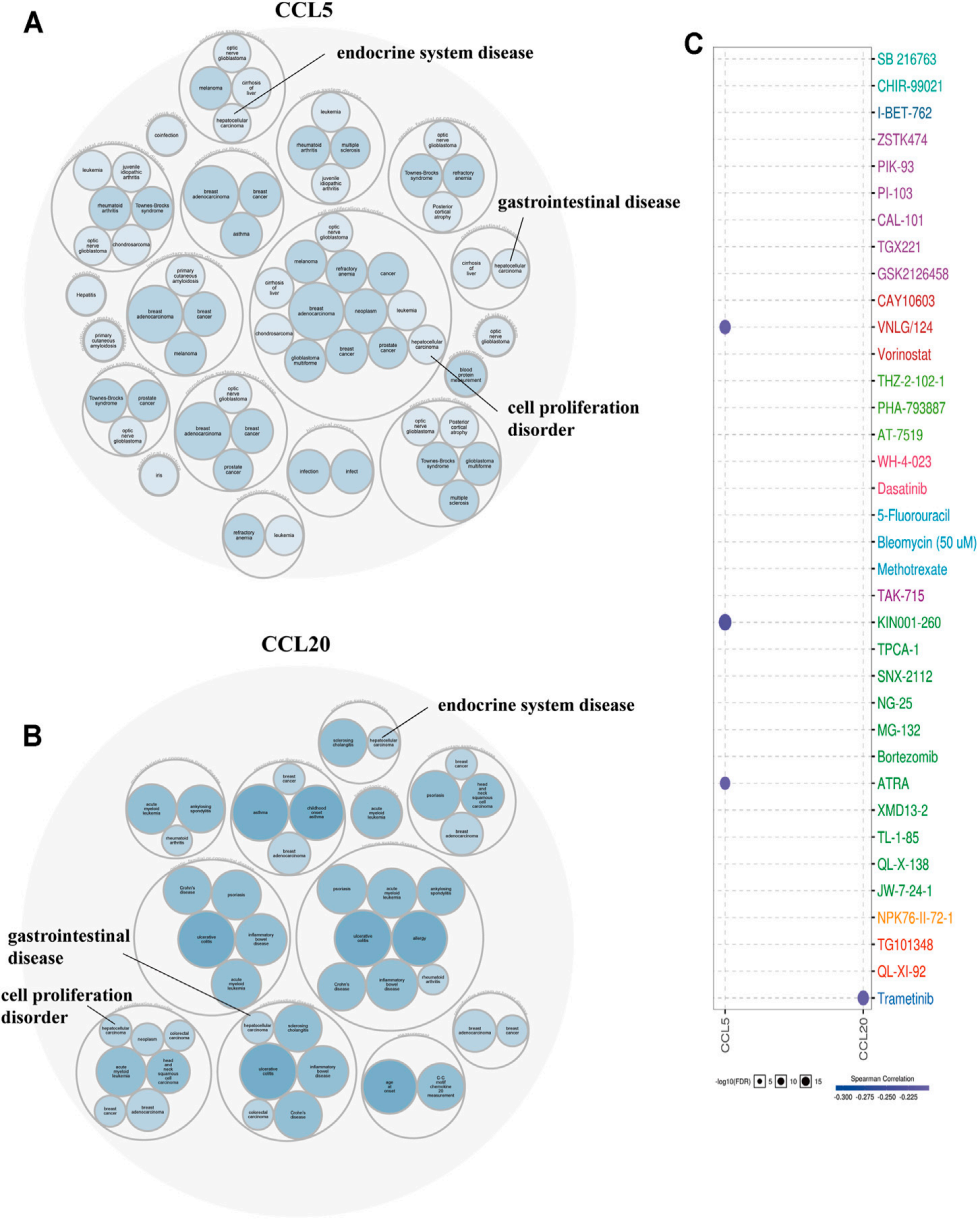
Disease correlation (Open Targets) and drug resistance analysis (GSCALite) of aberrantly expressed chemokines CCL. **(A)** Diseases related to CCL5, **(B)** Diseases related to CCL20. **(C)** Drug resistance analysis of chemokines CCL5 and CCL20.

To further determine the disease caused by the aberrant expression of CCL5, and CCL20, we performed an analysis based on the Open Targets database. The findings showed that these chemokines CCL were significantly related to gastrointestinal disease, endocrine system disease, and the disease of cell proliferation disorder (Figures 9A,B **)**. Among the GDSC database, the results indicated that there were 3 and 1 drugs or small molecules that could target the expression of CCL5 and CCL20, respectively (Figure 9C **)**. Among them, CCL5 was negatively regulated by VNLG/124, KIN001-260, and ATRA, while CCL20 was negatively regulated by Trametinib. These analyses provided potential strategies for the clinical treatment of aberrant chemokines CCL expression in patients with LIHC.

## Discussion

LIHC is considered the prototype of “inflammation-associated cancer,” because it usually originates in chronically inflamed tissue (Vucur et al., 2010). It is worth noting that chemokines CCL play a crucial role in inflammation and immunity, and they are also key mediators of cancer related inflammation being present at tumor site for pre-existing chronic inflammatory conditions but also being target of carcinogenic pathways (Mantovani et al., 2010). Previous studies have characterized the chemokines CCL play a vital regulatory role in tumor cell growth, tumor formation, invasion and metastasis (Chow and Luster, 2014; Yang et al., 2017). In addition, the role of chemokines CCL between the tumor microenvironment and cancer immunotherapy has gradually attracted the attention of researchers. However, the prognostic value and biological functions of chemokines CCL in LIHC urgently need to be further demonstrated.

Since the pathological stage of LIHC is critical to the prognosis of patients, we first explored the relationship between aberrantly expressed chemokines CCL and pathological stage in LIHC. Among the 18 aberrantly expressed genes in LIHC tissue compared with normal liver tissue (upregulation of *CCL5/8/11/13/15/18/20/21/25/26/27/28*; downregulation of *CCL2/3/4/14/23/24*), the transcriptional expression of *CCL25*, *CCL26*, and *CCL28* were obviously elevated with the aggravation of tumor malignancy while *CCL14* was reduced. Moreover, we have demonstrated that the high transcription levels of *CCL14* and *CCL21* in LIHC patients were significantly associated with better DFS, and high *CCL14* and *CCL23* transcription levels also brought longer OS. Therefore, all the findings proved that the differential expression of chemokines CCL was significantly associated with the progression and prognosis of LIHC.

Promiscuous interactions between chemokines and their receptors play a pivotal role in many pathological processes, including directly affecting tumor progression and therapeutic outcomes. To further clarify the interaction between these differentially expressed chemokines CCL in LIHC, PPI network and neighboring gene prediction were used for analysis. We found that the most significant functional interactions of these chemokines CCL included chemotaxis, cytokine activity, lymphocyte migration, and inflammatory response. As reported in previous studies, these functions could regulate immune cell infiltration and inflammation in the tumor environment (TME), which played an important role in tumorigenesis (Balkwill, 2004; Coussens and Werb, 2002; Koizumi et al., 2007).

In the tumor microenvironment, tumor cells can induce the recruitment and infiltration of various immune cells by expressing chemokines. We then performed a comprehensive exploration regarding the association between differential expression of chemokines CCL and immune cell infiltration. Accumulating evidence suggests that aberrant expressions of chemokines CCL regulate the recruitment of immune cells in tumors, such as CD8^+^ tumor infiltrating T cells and macrophages, which may ultimately affect the clinical outcome of cancer patients (Dangaj et al., 2019; Lee et al., 2017). As shown in our results, there was a positive or negative correlation between the expression of chemokines CCL and the infiltration of the six immune cell types, B cells, macrophages, neutrophils, dendritic cells, CD4^+^ and CD8^+^ T cells. What’s more, the multivariable Cox proportional hazard model indicated that the expressions of CCL2, CCL5, and CCL20 may be potential risk factors affecting the clinical outcome of patients with LIHC. Furthermore, chemokines have been proven to be important regulators of immune cell infiltration and immune checkpoint blocking efficacy in the TME (House et al., 2020; Karin, 2018). Similarly, our research suggested that CCL2 and CCL5 were positively correlated with immune checkpoints such as PD-1, PD-L1, and CTLA-4, while CCL20 was also positively correlated with PD-1 and CTLA-4.

In the analysis of potential transcription factor targets of the differentially expressed chemokines CCL, a total of 9 key transcription factors (RELA, REL, NFKB1, STAT1/3/6, IRF3, SPI1, and JUN) were involved in the transcriptional regulation of chemokines CCL (including *CCL2, CCL3, CCL4, CCL5, CCL11, CCL13, CCL20, CCL21*). RELA post-translational modification, particularly phosphorylation, has been proven to be critical for abnormal NF-κB activation (Lu and Yarbrough, 2015). Moreover, NFKB1 has been reported to play a crucial role in attenuating the activation of the NF-κB signaling pathway, potentially revealing a new therapeutic target in inflammatory diseases and cancer (Cartwright et al., 2016). Studies have also shown that phosphorylation of cellular JUN regulates the secretion of chemokines by macrophages in LIHC-bearing liver, and affects the recruitment of regulatory T cells and tumor progression (Hefetz-Sela et al., 2014). Therefore, these data indicate that the chemokine CCL mediates the progression of LIHC disease through a variety of signaling pathways, which also provides potential targets for clinical treatment strategies.

To more clarify the mechanism of the differentially expressed chemokines CCL in LIHC, we performed GO functional enrichment and KEGG signal pathway enrichment analyses. Among them, functional enrichment analysis showed that the aberrant expressed chemokines CCL and their similar neighboring genes were mainly related to chemokine-mediated signaling pathway, chemotaxis, NF-κB signaling pathway, TLR signaling pathway, and TNF signaling pathway. The findings were consistent with the previous studies demonstrating that chemokine-mediated signaling pathways play vital regulatory roles in oncogenic processes including tumor proliferation, apoptosis, epithelial–mesenchymal transition, immune evasion, and metastasis (Bian et al., 2019; Bikfalvi and Billottet, 2020; Zhuang et al., 2018). Furthermore, both the NF-κB signaling pathway (Dolcet et al., 2005) and TLR signaling pathway (Patra et al., 2020) have been confirmed to be involved in a variety of pathological processes in tumor progression.

In this study, we further verified that CCL5 and CCL20 were significantly upregulated in both HCC cell lines and tumor tissues compared with normal controls by Western blot and IHC, which were consistent with the transcriptional levels. We then paid attention to the TNF signaling pathway regulated by CCL5 and CCL20. TNF, the trigger of tumor cell apoptosis, was a major mediator of inflammation and immunity in tumor microenvironment, and there were strong evidences that this cancer-related inflammation contributes to the proliferation of malignant cells, stimulates angiogenesis and metastasis, regulates immune response, and affects clinical treatment strategies (Chen and Goeddel, 2002; Mantovani et al., 2008; Balkwill, 2009). Interestingly, TNF could also induce the activation of transcription factors c-jun and NF-κB, which ultimately activated JNK kinase activity and NF-κB signaling pathways, respectively (G. Chen et al., 2002). Besides, the disease susceptibility prediction found that aberrant expressed CCL5 and CCL20 were mainly related to gastrointestinal disease, endocrine system disease, and the disease of cell proliferation disorder (including LIHC). CCL5 expression was additionally negatively regulated by some small-molecule drugs, including VNLG/124, KIN001-260, ATRA, while CCL20 was negatively regulated by Trametinib. It is worth noting that immunotherapy has revolutionized the treatment of cancer in recent years. It has been reported that the upregulation of CCL5 may promote immune evasion through recruiting Treg cells into tumor lesion and anti-PD-L1 treatment could enhance CCL5-mediated anti-tumor effects in pancreatic ductal adenocarcinoma (Wang et al., 2020). We, therefore, demonstrated that CCL5 was positively correlated with PD-1, PD-L1, and CTLA-4, whereas CCL20 was positively correlated with PD-1 and CTLA-4. The collective evidence supports the potential of chemokines CCL as prognostic markers and clinical treatment targets for patients with LIHC.

Although bioinformatic approaches have been widely used in the research of human diseases, there are some limitations in our study. First, the results of gene expression presented by database analysis (such as Oncomine) are mainly mRNA levels. However, gene expression is regulated at multiple levels, including epigenetics, transcription, translation and post-translation. Especially in tumor-related research, we should also pay attention to the impact of gene protein expression levels on disease progression. Secondly, bioinformatics analysis is relatively mature today, which provides a broader perspective for the potential mechanisms and therapeutic targets of disease progression, whereas the experimental verifications on this basis are necessary, which is also the most critical limitation of our research. Finally, small sample size verification in this study is far from sufficient, and we look forward to large-sample clinical cohort studies and biological validation *in vitro* or *in vivo* that can be performed in the future to further verify the mechanism of LIHC progression.

In summary, our research findings hope to provide potential prognostic biomarkers for LIHC patients, and provide new insights for their therapeutic targets and clinical medications, which may help guide treatment strategies.

## Data Availability

The datasets presented in this study can be found in online repositories. The names of the repository/repositories and accession number(s) can be found in the article/supplementary material.

## Abbreviations

BP, biological process; CC, cellular component; CCL, chemokine (C-C Motif) ligand; CTRP, Therapeutics Response Portal; CTLA-4, cytotoxic T-lymphocyte-associated protein 4; DFS, disease-free survival; GEPIA, Gene Expression Profiling Interactive Analysis; GEO, Gene Expression Omnibus; GDSC, Drug Sensitivity in Cancer; GO, Gene Ontology; HCC, hepatocellular carcinoma; KEGG, Kyoto Encyclopedia of Genes and Genomes; LIHC, liver hepatocellular carcinoma; MF, molecular function; NTA, Network Topology-based Analysis; NF-κB, nuclear factor kappa B; OS, overall survival; PD-L1 (CD274), programmed cell death-ligand 1; PPI, protein–protein interaction; TCGA, the cancer genome atlas; TF, transcription factor; PD-1(PDCD1), programmed cell death protein 1; TLR, toll-like receptor; TNF, tumor necrosis factor.
